# Supplementary figures and images for: Feedback information transfer in the human brain reflects bistable perception in the absence of report
Source: PLoS Biol. 2023 May 8;21(5):e3002120. doi: 10.1371/journal.pbio.3002120 (PMC10194963; doi:10.1371/journal.pbio.3002120)

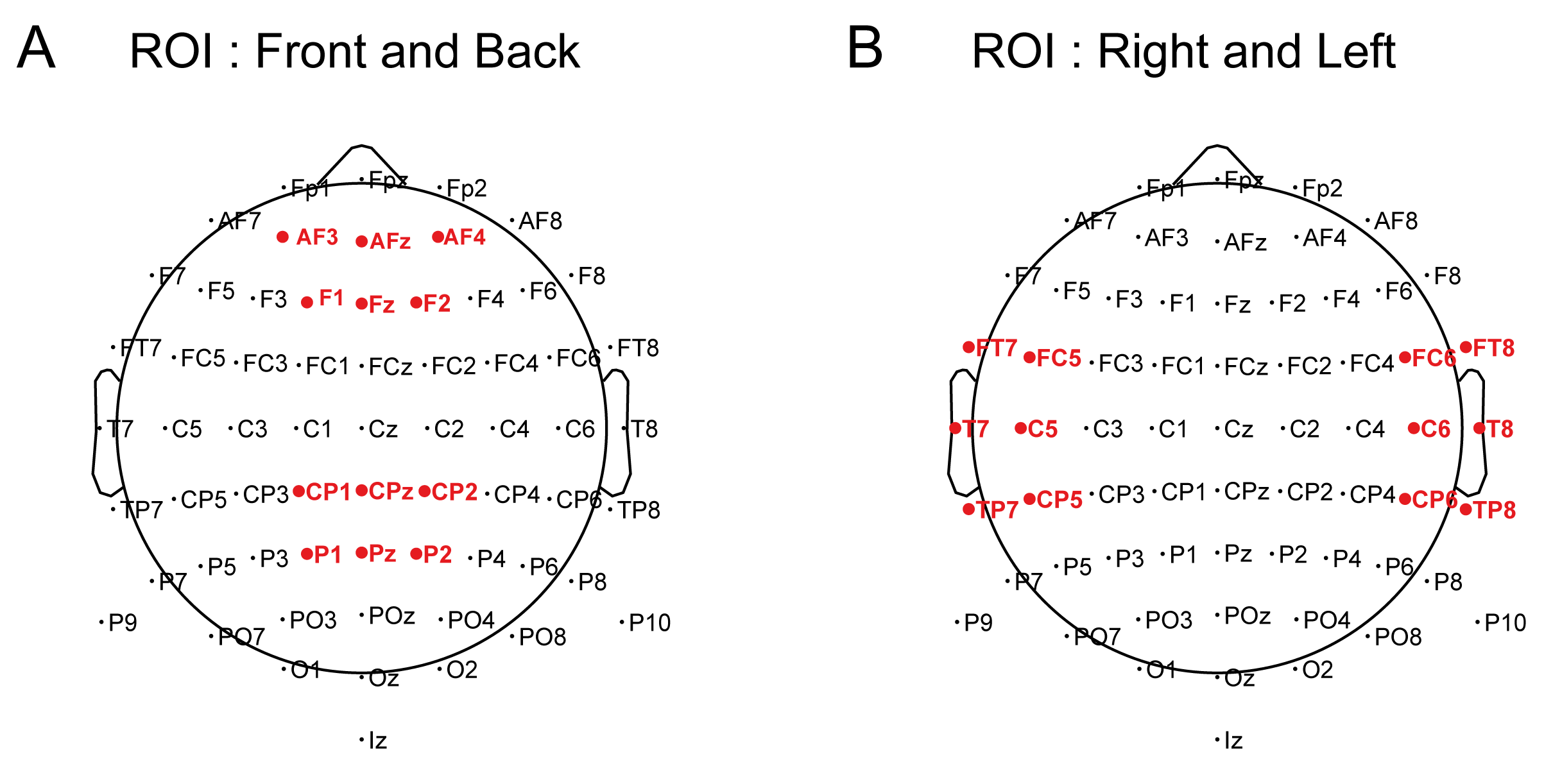

Supplement: S1 Fig — Electrodes included in the (A) front and back ROIs, and (B) right and left ROIs. (TIF) [file pbio.3002120.s001.tif]

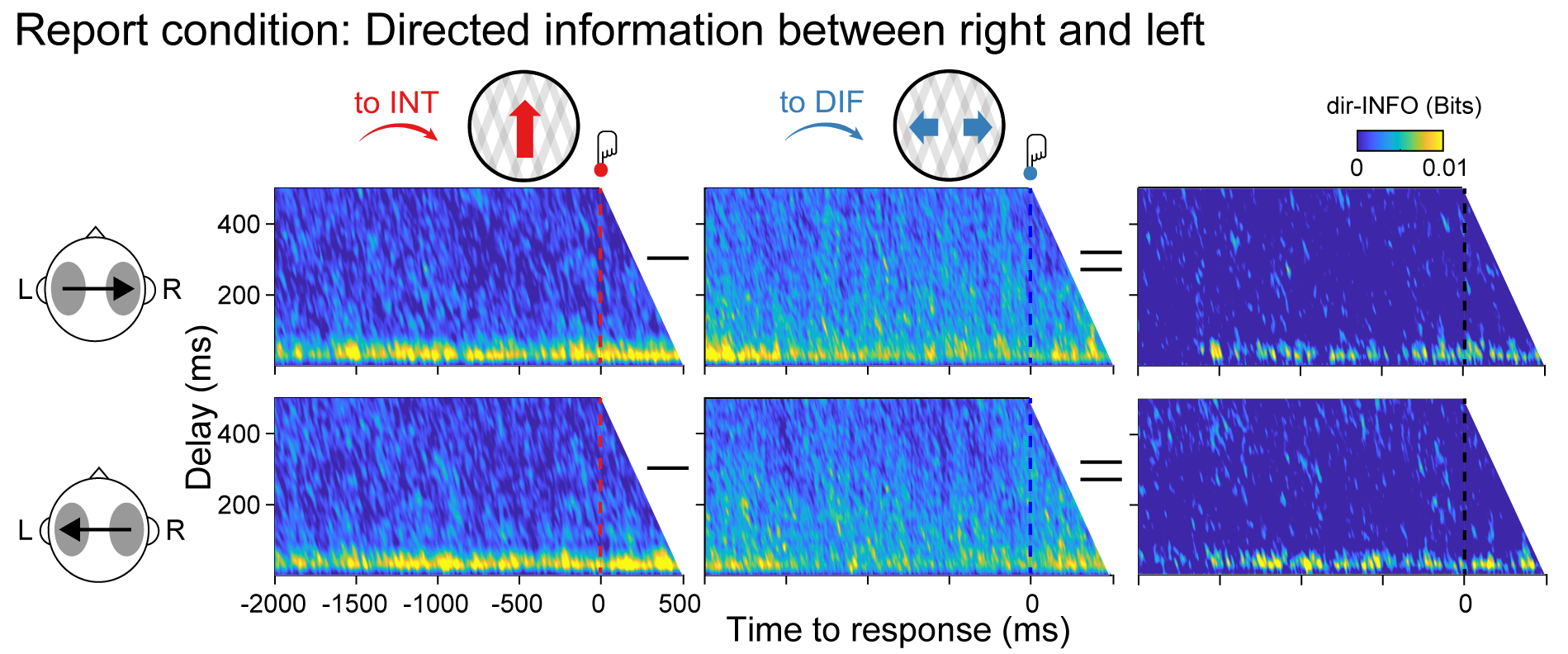

Supplement: S2 Fig — Group-level dir-INFO between right and left ROIs (upper row) and between left and right ROIs (lower row) when moving plaids are reported as integrated (to INT; red color), reported as differentiated (to DIF; blue color). No significant clusters were found. (TIF) [file pbio.3002120.s002.tif]

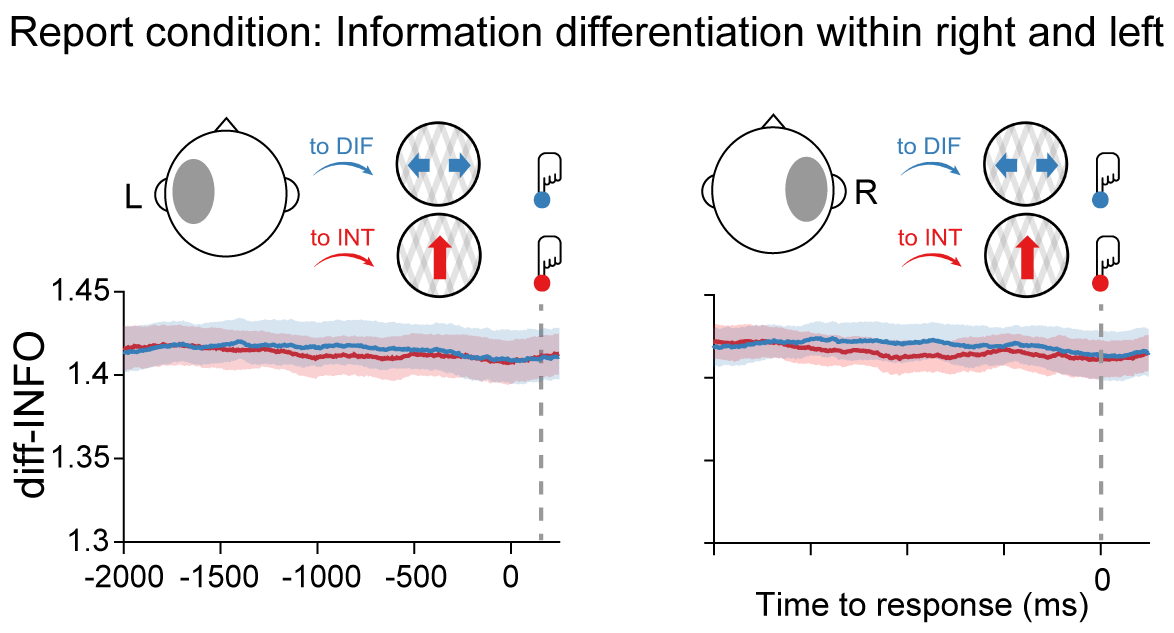

Supplement: S3 Fig — Group-level diff-INFO within the left temporal ROI (left panel) and right temporal (right panel) when moving plaids are reported as differentiated and reported as integrated. No significant clusters were found. (TIF) [file pbio.3002120.s003.tif]

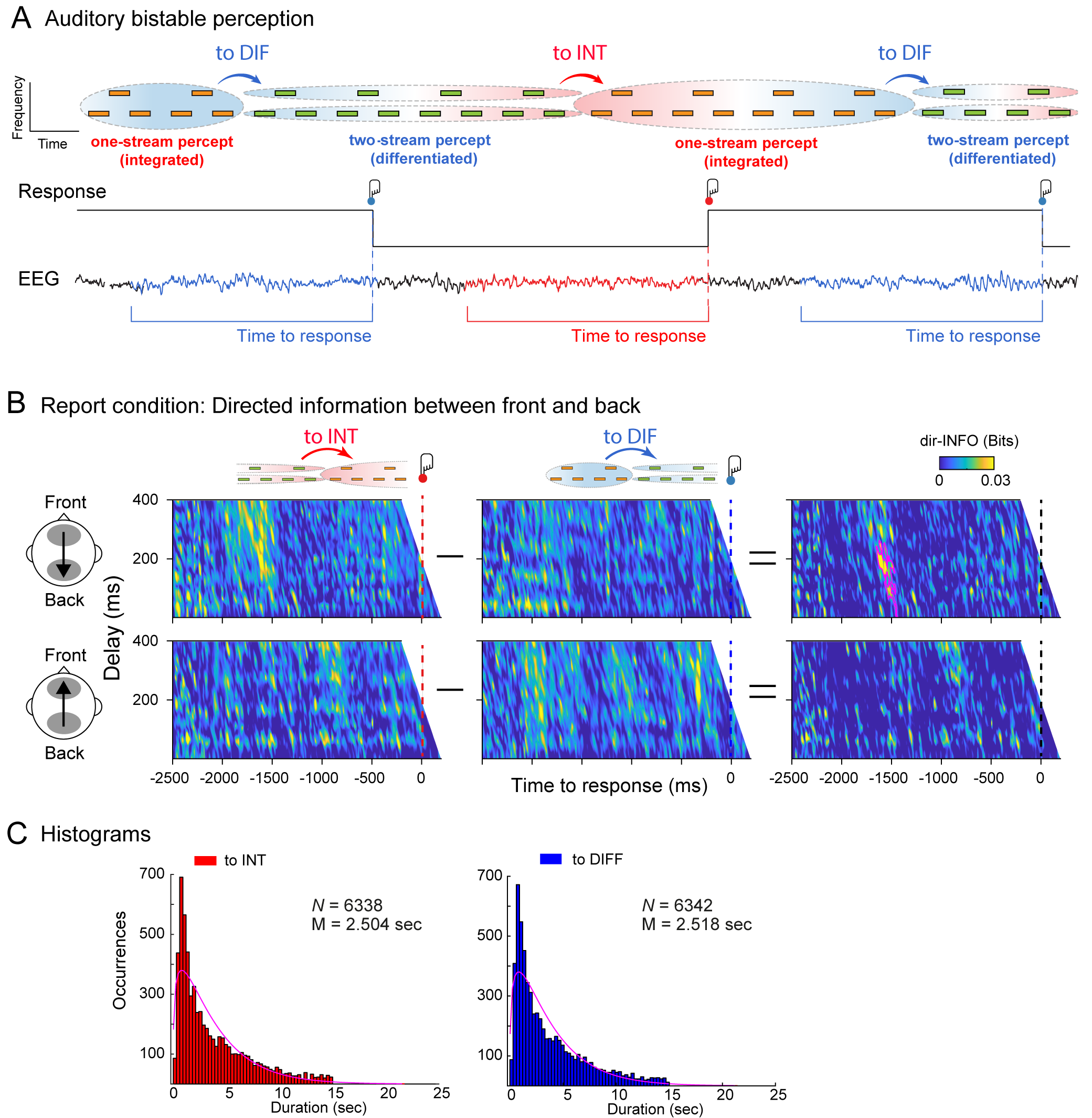

Supplement: S4 Fig — (A) Phenomenology during auditory bistability. Participants (N = 29) listened to an ambiguous auditory stream that is experienced either as one auditory stream (integrated percept; red arrow) or two auditory streams (differentiated percept; blue arrows). Perceptual transitions occur either in integrated to differentiated direction (to DIF; blue) or in the differentiated to integrated direction (to INT; red). Middle row: Behavioral responses during the task. Participants pressed one button when perceiving that the integrated percept had fully changed into the differentiated percept (red button) and another button when perceiving that the differentiated percept had fully changed into the integrated percept (blue button). Bottom row: dir-INFO analyses for EEG signals before the button press. (B) Group-level dir-INFO from front to back ROIs (upper row) and from back to front ROIs (lower row) when the auditory stream is reported as integrated (to INT; red color), reported as differentiated (to DIF; blue color), and the cluster-based permutation tests between the two. Note that the significant cluster is observed approximately 1,500 ms before the response, indicating that a perceptual switch has taken place, which is slightly longer than for our visual bistability results reported in Fig 2A. This minor time difference is likely due to the fact that perceptual states alternated slower in the auditory compared to the visual version of the task. (C) Histograms for perceptual switches in the report condition locked to the button response. (TIF) [file pbio.3002120.s004.tif]
